# Job autonomy and work-life conflict: A conceptual analysis of teachers’ wellbeing during COVID-19 pandemic

**DOI:** 10.3389/fpsyg.2022.882848

**Published:** 2022-07-25

**Authors:** Sonia Khawand, Pouya Zargar

**Affiliations:** Department of Business and Economics, Girne American University, Kyrenia, Cyprus

**Keywords:** job autonomy, wellbeing, work-family conflict, work-life balance, qualitative analysis, academic staff, COVID-19

## Abstract

With the shift toward online environments due to COVID-19 pandemic, particularly for educational sector, employees’ performance has been affected by an array of different factors. Personal aspects as well as organizational focus on individuals’ wellbeing are the main focus of this study through inclusion of job autonomy and work-life conflict alongside other factors, such as informational support that can aid academic staff regarding their wellbeing during times of crisis. In response to the *effects of COVID-19 on employees,* this study aims to provide tangible data to protect university teachers during crises and establish key points that can improve their wellbeing. For this purpose, we used interviews to provide in-depth understanding of the subject. A total of 16 teachers as interviewees have provided qualitative data that was analyzed with MAXQDA (thematic network approach). This study highlights the importance of work-life conflict and vitality of job autonomy on academic staffs’ performance and overall wellbeing through a conceptual analysis. We emphasize the role of organizations in maintaining a work environment where university teachers’ wellbeing is prioritized and various elements such as training and support are used to help stabilizing work-life balance. The current findings can be beneficial for both scholars and decision-makers in schools and universities to enhance elements of *remote work* for their staff.

## Introduction

Amid the occurrence of the global pandemic of COVID-19, majority of industries were severely affected on a global scale with education sector having to face an abrupt and forced change to maintain its flow. Various studies have noted the enormous psychological, physical, economic, and other impacts of the pandemic (e.g., Nande et al., 2021; Schubert et al., 2021; Silva et al., 2021; Zöllner and Sulíková, 2021). The current research follows a string of new studies that address the psychological impacts of COVID-19 pandemic on employees. Specifically, this study focuses on academic staff due to abruptness and short period of time that they had to adjust to new online systems. There have been studies that addressed academic staff and psychological elements during the current pandemic (e.g., Telyani et al., 2021). However, the notion of job autonomy and its important influence on academic staff has been studied in the extant literature (e.g., Song et al., 2011). The current research aims to provide an in-depth understanding of job autonomy and its effects on academic staffs’ performance, and overall wellbeing. In this study, we argue that job autonomy and support from the management/organization play a key role in university teachers’ wellbeing, especially psychological wellbeing during times of crises such as the COVID-19 pandemic.

Following what was noted above, the current study highlights the importance of understanding psychological effects of COVID-19 pandemic among academic staff due to their intense workload, and the mixture of their work with their families in an abrupt manner. Notably, the literature of the subject is scarce as the status quo has occurred in a manner, which did not allow the education sector to be prepared for this shift (e.g., Silva et al., 2021; Telyani et al., 2021). While some studies have suggested deeper insights regarding the topic at hand through self-report data of individuals and/or their acquaintances (e.g., Sarwar et al., 2021), others have suggested using different theories, and more relevant to the current case, understanding daily experiences of individuals during the pandemic of COVID-19 to comprehend the effects of having a high demanding work mixed with home duties (Darouei and Pluut, 2021). In this respect, job autonomy is an important matter that can enable academic staff to better conduct and balance their work and life responsibilities. This is in line with a recent string of research that addresses the notion of the impacts of pandemic on employees (e.g., Bevan. 2020; Case, 2020; Darouei and Pluut, 2021). Hence, this research addresses the gaps found in the most recent literature (as cited in this section) regarding teachers’ wellbeing in general and importance of autonomy and work-life conflict in particular through a conceptual analysis.

As the core concept of this study is to look into important elements influencing wellbeing of teachers during COVID-19 pandemic, it is also vital to highlight the fact that to establish the current framework, a rigorous review on the literature has been conducted using the criteria of most recent and/or most relevant terms (e.g., synonyms) and findings from various databases (e.g., Clarivate, Scholar Google, and EBSCO). Hence, a number of theories and findings relevant to the current case have been gathered to support the arguments of this research. Emphasizing wellbeing and job autonomy, this research argues that provision of job autonomy to academic staff (i.e., teachers) has a vital role in their job satisfaction, performance, work-life conflict, work-life balance, and overall wellbeing (Winefield et al., 2014; Rasool et al., 2019, 2022; Schall, 2019; Darouei and Pluut, 2021; Sarwar et al., 2021). With regard to the prior findings in the literature and various theoretical frameworks that support the aforementioned statement, this study argues that university managers and decision-makers can significantly help their teachers to maintain a balance in their life, which, in turn, provides comfort and establishes an environment, where care and support are provided. Arguably, this can be achieved through autonomy, support, and focusing on the quality of life of university teachers. Notably, due to the fact that teachers have had to change their work environment upon the pandemic, lack of social interactions and other psychological elements (e.g., work stress) is included within the scope of current research. Using coping strategies such as sports, leisure activities, humor, spirituality, meditation, and personal time (e.g., relaxation, art, and music) can be escape routes from stress and anxiety induced by work for teachers. The following sections of this research include reviewing the extant literature regarding recent and relevant findings, and theoretical frameworks and settings, followed by the description of the current undertaken methodology, reports of findings, and implications of the study. Lastly, limitations and recommendations for future scholars are provided.

## Literature review and theoretical setting

The extent to which individuals perceive autonomy is a vital factor for their wellbeing. This has been noted in a number of studies from different disciplines, while teachers have been regarded as a group highly focused by scholars (e.g., Winefield et al., 2014; Lases et al., 2018; Collie and Martin, 2020; Collie, 2021; Zöllner and Sulíková, 2021; Syrek et al., 2022). It has been noted that wellbeing can be categorized into professional and personal aspects. While the former refers to teamwork and experiences gained from workplace and colleagues, the latter addresses individual features that enable handling work and life in a balance (Lases et al., 2018). Autonomy is described as the level of control possessed by employees regarding the conduct of “their tasks” (Hackman and Oldham, 1976; Breaugh, 1985; Khoshnaw and Alavi, 2020). This factor can have significant and vast impacts on a number of organizational and behavioral elements such as commitment, satisfaction, work-life balance, stress and burnout, engagement, and performance.

Within the premises of *self-determination theory*, job autonomy provides more motivation and personality for individuals, positively affecting their outcomes in personal and professional manners (Deci and Ryan, 1985; Deci et al., 2017). The theory is linked to job autonomy through triggering intrinsic motivation. The level of control an academician has on their role is a vital element in this respect that is categorized into psychological (autonomy), work-life setting, and relational aspects (e.g., lack of social engagement). Notably, for the case of the current study that addresses teachers as academic staff, the lack of interaction with peers, students, and managers in a sudden form has been reported to be a matter of importance. Various factors such as stress, anxiety, engagement, motivation, performance, and turnover have been witnessed during the pandemic. As a career that has deep impacts on generations of a society, and collectively the world, teachers’ wellbeing has been a topic of vitality among scholars and practitioners.

In addition, *role theory* (Biddle and Thomas, 1966) explains how individuals can have conflicts due to various roles that they carry out. The performance of individuals can greatly reduce in their roles, as adequate focus and strength do not match the requirements of different roles, and hence, inter-role conflict can appear (Jawahar et al., 2012; Adisa et al., 2021). This study emphasizes the conflicts among roles of teachers during the global pandemic of COVID-19 specifically work-life conflict. From the start of pandemic, a considerable number of researchers have studied different aspects related to the context of this study. However, the current research uses a number of theories and settings into account and further provides an in-depth analysis of the gathered data, which contributes to the literature and decision-makers within the education sector. Job autonomy has been regarded in the literature to be a major determinant for individuals in handling their roles in life. Thus, having a better approach toward challenges in their personal aspects of lives. Organizational support and various Human Resource Management (HRM) practices also have been reported to be influential in this context (Rasool et al., 2019, 2022; Novitasari et al., 2020; Vaziri et al., 2020; Andrade and Petiz Lousã, 2021; Ghislieri et al., 2021).

In accord with what was noted, *the Job Demands Resources* (JD-R) model (Bakker and Demerouti, 2007) also falls within the scope of the current study that explains how the availability of resources leads to individuals meeting the requirements of their jobs (Gajendran et al., 2015). In this respect, having freedom and control to conduct work-related tasks for teachers can enhance the notion of autonomy and determination. As the demands of education sector are high, this model is used in recent findings to explain work-life balance, and other psychological aspects of academic staff (e.g., Sarwar et al., 2021). Decisions regarding location, or details of tasks, are important elements that can reflect autonomy for teachers. In this sense, work-life conflicts arise when there is an incompatibility between the two roles. As noted earlier, the factors that are related to the current context are complex by nature. Nevertheless, work-life conflict is related to a number of constructs that include but are not limited to, job autonomy, demands, supervisor and organizational support, burnout, engagement, motivation, and wellbeing. There have been numerous studies conducted in the extant literature that support the aforementioned argument (e.g., Greenhaus and Beutell, 1985; Bentley et al., 2016; Chambel et al., 2017; Schall, 2019; Sarwar et al., 2021; Rasool et al., 2022; Zaman et al., 2022). However, majority of studies have examined different sectors (e.g., telecommunication, and IT). As a high demand sector, teachers should be equipped with knowledge, information, usage, coping and management strategies, and other characteristics. This further increases the importance of studies that can provide a better approach for fostering teachers’ needs. Notably, there are various studies that note a difference between genders regarding stress, anxiety, and behavioral issues caused by COVID-19 pandemic, which implies the importance of understanding different factors related to this context (e.g., Kirkman, 2020; Sampson, 2020; Stevens, 2020).

In the context of work-life conflict, role theory is also used to describe the positive impacts of remote work through decreased inter-role issues aforementioned earlier. This has also been linked to stress and job satisfaction in recent findings (e.g., Hong et al., 2021; Nemteanu et al., 2021; Nemteanu and Dabija, 2021). The literature has also noted that women are more susceptible to have their roles conflict with one another due to the variety of tasks that they perform in daily life, especially those with children. Notably, this factor has been reported to be important regardless of their response (to choose work over family role; e.g., Novitasari et al., 2020; Adisa et al., 2021). The research on job satisfaction has shown that remote settings scored higher by employees in IT or telecommunication. However, for educational sector, the noted shift posed by the pandemic forced the academic staff across the world to adjust to online systems and remote work settings. While flexibility increases in such settings, other aspects such as autonomy, work-life balance, time pressure, and other stressors can have varying effects for different individuals.

Managers and leaders constantly seek strategies and practices that can enhance work-life balance for their staff. Within the education sector, this is relatively less examined, when compared to other sectors. Furthermore, individual characteristics such as proactivity, resilience, neuroticism, and other psychological capabilities, and resources are vital for better managing work-life balance. This becomes more vivid and important, when facing crises that trigger stress, anxiety, and can have varying demands that require adjustment (Greenhaus and Allen, 2011; Morganson et al., 2014; Wayne et al., 2016, 2020; Rasool et al., 2020; Sarwar et al., 2021). Individual characteristics are determinant factors regarding perception and emotions toward work-life balance. As both work and family are domain-based in terms of demands and resources, the context of current research fits JD-R model premises linked to work-life conflicts. This study looks at the impacts of COVID-19 pandemic on university teachers which their roles in both personal and professional aspects are in line with JD-R model, as work-life conflict is a hindering element for wellbeing. It is important to highlight that due to the high intensity of academic work, more time and energy are required for conducting tasks (e.g., grading, assignments, etc.), which causes an imbalance between work and life realms (Winslow and Davis, 2016; Sarwar et al., 2021). Moreover, academic studies or research are beyond the norms of work as they can last for long periods (Curtis, 2004). The process of research and teaching are constant and paired with one another, further increasing negative impacts on balancing ones’ life.

For the case of current research, as university teachers were forced to work with newly designed platforms, resources were low. Additionally, lack of proper information and training for using online platforms as well as access to adequate connections are among the challenges for the education sector upon the occurrence of COVID-19 pandemic. This further negatively impacts the balance between work and life, particularly for academic staff (i.e., teachers; Peeters et al., 2005; Marais et al., 2014). Subsequently, this has diminishing effects on the overall wellbeing of academicians (Hong et al., 2021; Sarwar et al., 2021). In addition, stress, being alone, time pressure, and anxiety have been reported by recent studies to be highly important regarding working from home during the pandemic and its relationship with wellbeing, and work-life conflict (Delanoeije and Verbruggen, 2020; Darouei and Pluut, 2021).

Time, energy, and focus are among the resources that are limited for each individual. Work-life balance is severely affected for teachers during the pandemic, and as teaching is an intensive domain, it drains the resources of individuals. This can lead to work–family conflict, stress, anxiety, and less engagement and performance, hindering teachers’ wellbeing. The extent of work during intense times (e.g., exams, and registration period) can drain more of personal resources, which for teachers working from their homes this is considered a significant demand from their job (Delanoeije et al., 2019; Rasool et al., 2021; Syrek et al., 2022). This can especially be seen regarding the time-consumption rate of teaching tasks that require focus and attention outside the teaching time. This is further linked to the resource loss spiral as mentioned earlier, as depletion of one resource will create vulnerability for individuals (Hobfoll, 1989; Darouei and Pluut, 2021). Notably, the performance of individuals can be affected in the following days after having a work–family conflict. Therefore, the current research focuses on job autonomy as a major element that enables academic staff (i.e., university teachers) to better handle their roles at work and at home during COVID-19 pandemic. Work engagement, burnout, exhaustion, stress, and anxiety as well as other underlying factors are aimed to be highlighted through the conduct of this research, following the recent findings (e.g., Wayne et al., 2020; Collie, 2021; Hong et al., 2021; Rasool et al., 2021; Telyani et al., 2021).

*Compensation theory* addresses the relationship between family, work, and life, which is an inverse correlation (Gerhart et al., 2003; Armstrong et al., 2015). Within the same scope, *conflict theory* (Bartos and Wehr, 2002) describes the incompatibility between demands of one domain with the other, which undoubtedly leads to work-life conflict. Both aforementioned theories fall within the current context as the former implies a dissonance among work, family, and life, and the latter further emphasizes their incompatible nature. For university teachers during the pandemic, the relationship between work and life has been mixed and due to the toll that teaching takes on individuals, conflicts among domains of life are observed. In turn, these impacts negatively influence job satisfaction and performance, which further diminishes the work-life balance (Schubert et al., 2021). Another relevant theory to the current context is *Action-Regulation theory* that addresses behaviors of individuals in workplace with regard to their goals (Frese and Zapf, 1994). In this respect, behaviors are psychologically regulated based on a sequence of cognitive processes (i.e., setting goals, planning, observation and tracking, and feedback process; Zacher and Frese, 2018; Schubert et al., 2021). The extent of flexibility within patterns of action, intellectual level, meta-cognitive level, and sensorimotor levels are within the hierarchy that regulates actions of individuals, which further connects mental pictures of actions, results, boundaries of conditions, and linkage among actions: heuristics, meta-plans, strategies, routine actions, and both unconscious and conscious schemata (Frese and Zapf, 1994). As individuals complete their work-related tasks through sequential and hierarchical actions and regulations, modification and adjustment of mental and behavior aspects can be achieved. Various skills and capabilities are then used for seeking development and achievement of goals, which translate into higher degrees of performance (Zacher and Frese, 2018).

Referring to action-regulation theory, the setting of work should be in a manner that provides complexity, and control so that coping mechanisms with regard to the environment are effective. In this sense, stressors that can arise from work are to be minimized for optimum results (Zapf, 2002; Hacker, 2003; Schubert et al., 2021). The job of teachers is challenging by nature and is parallel with control, which establishes both requirements and possibilities of regulation. In turn, this leads to decreased strain and therefore can have a positive impact on the overall wellbeing of teachers. This is further linked to JD-R model, which emphasizes job autonomy (Schubert et al., 2021). The vitality of this becomes more vivid for the case of teachers due to the high demands of their work. Notwithstanding that during COVID-19 pandemic, the demands have been combined with other stressors (e.g., work-life balance, conflict, anxiety, and stress).

Within the context of this study, work-life balance, when in a satisfactory level, can yield in happiness, increased wellbeing (e.g., healthy lifestyle), and better performance in the professional domain. While the concept of wellbeing is described by various scholars, we consider both physical and psychological wellbeing for university teachers that include autonomy, good bonds with others (e.g., peers, family, and friends), having purpose, acknowledging ones’ potential, accepting ones’ self, “environmental mastery,” being capable of fulfilling goals, happiness, and life satisfaction (Ryff, 1989; Diener and Suh, 1997; Foresight Mental Capital and Wellbeing Project, 2008; Dodge et al., 2012). Vivid for teachers, this factor carries a major role in terms of quality of life in an overall sense (Galanti et al., 2021; Tewal et al., 2021). Considering university teachers’ specific tasks (i.e., lecturing, conducting research, advisory tasks for students, administrative tasks such as grading and university platform handling), a number of elements can impact work-life balance that are namely, (a) institutional improvements for learning; (b) increased tasks or maintaining an updated knowledge; and (c) variations in job demands, which directly affect all domains of life (Pasamar and Cabrera, 2013). Consequently, performance is hindered and wellbeing is jeopardized. This impact is linked to the aforementioned theories as work collides with daily life. Younger generations of teachers have shown more susceptibility toward having their work-life balance diminished. This can be related to their goals (e.g., financial security, independence, image, and experience). Importantly, when a high demanding job such as teaching enters daily life, and is combined with restrictions (e.g., social interactions), it can greatly impact wellbeing of academic staff (Neneh, 2017). This research focuses on the role of job autonomy and organizational role (i.e., informational and supervisor support) to provide a better understanding on how teachers can be protected.

According to new studies in the same context, stressors that teachers face are categorized into three inter-related aspects that are namely, coping, competence, and context (3C Theory of Teacher Stress). Coping is referred to coping strategies and methods that are deployed by an individual for better handling of their tasks, and enhance emotional and psychological status (e.g., sports, yoga, leisure, etc.). Competence can be described as the level of know-how possessed by teachers regarding deliverance of knowledge; and context is the collective of policies, methods, practices, and administrative support (Herman et al., 2020). It is important to highlight that the scope of the aforementioned stressors expands to all aspects of life. With the pandemic, environmental or external stressors have rapidly increased as health (mental and physical), safety, activities, transportation, and other means plummeted. Negatively impacted work-life balance, and added demands of job alongside conflicts that can arise, are among other important elements that should be taken into consideration. This can be more explicit for the case of female teachers as task overload and increased conflicts can require more coping mechanisms, resources, and tolerance, which are limited for individuals (Neneh, 2017; MacIntyre et al., 2020).

Coping strategies can be categorized into a number of groups that are generalized into approach, avoidant, and neither-approach-nor-avoidant (see Carver 1997 as cited by MacIntyre et al., 2020). Denial and distraction are among avoidant groups while looking for changing the stressor or acceptance fall within approach strategies. In this sense, using humor in the face of events and religion (coping through spirituality) fall in the domain in between the aforementioned approach and avoidance. These combined with competence and context during the pandemic have given rise to various stressors simultaneously, negatively impacting performance, wellbeing, and balance in the lives of all humans, and particularly, teachers as the case of the current study. Negative emotions are the fruit of failed coping strategies. For teachers, this can lead to exhaustion, anger, denial, withdrawal, substance usage, aggression, burnout, depression, and other “maladaptive behaviors.” In this context, it is important to note that the aforementioned behaviors can impact students through contagion of stress (Oberle and Schonert-Reichl, 2016; Herman et al., 2020; MacIntyre et al., 2020). As lack of balance can have severe impacts on individuals’ life, it has been reported that female teachers have shown a tendency toward flexible schedules as a mechanism to cope with time demands of their work in Saudi-Arabia (Al-Alawi et al., 2021). Both performance and productivity have been reported to be diminished due to work-life and work–family conflicts. Hence, having a balance between work and life carries a major role in performance.

Organizational support in terms of providing sufficient and adequate resources of information, and tools, as well as the role of supervisors in the provision of aid for academic staff have been reportedly noted by scholars. These factors are similar to job autonomy in the context of which individuals require resources and balance for better performing in their roles (Charoensukmongkol and Phungsoonthorn, 2021; Oubibi et al., 2022; Rasool et al., 2022). Similar to other careers, teachers carry a number of responsibilities. Engagement and involvement with educational approach of the institutions as well as teaching methods are among key aspects of teachers’ overall performance. Teachers with more engagement have shown higher rates of productivity, loyalty, and better deliverance of knowledge to their students (Oubibi et al., 2022). Supervisors and organizations can provide the necessary means for teachers’ wellbeing, and work-life balance, which lead to positive outcomes such as higher engagement levels (Collie, 2021). The current research focuses on the role of organizations and subsequently, supervisors in fostering an atmosphere during the COVID-19 pandemic for teachers, where they can have more involvement and engagement. This, in turn, can lead to enhanced performance, which is of necessity during this crisis as both teachers and students’ wellbeing is affected. We emphasize the provision of autonomy, support, and other practices that can benefit teachers as individuals.

## Methodology

The current research uses specific criteria regarding its context and approach. Using an in-depth interview approach toward the subject at hand, this research aims to provide a thorough understanding of the important elements hindering wellbeing of teachers during COVID-19 pandemic. Considering the aims and scope of this research, descriptive phenomenology is used to highlight the essence of the notion through the eyes of those experiencing the phenomenon. In direct interaction (researcher and object), interviewees are regarded as representatives of their own world. As the research aims to understand the experiences of teachers during times of lockdown and pandemic, and its effects on their wellbeing, this method is deemed appropriate (Starks and Trinidad, 2007; Wojnar and Swanson, 2007; Willig, 2013). In this respect, job demands are used as a concept that participants are questioned on (i.e., quantitative, emotional, and cognitive demands; Van Veldhoven and Meijman, 1994; Sarwar et al., 2021). This established the setting in which interviewees are prompted to provide details regarding the demands of teaching during COVID-19 pandemic. Furthermore, job resources were involved through addressing job autonomy, collegial and supervisor support, and windows for professional development. Moreover, questions addressing work-life balance were derived from various sources (e.g., Currie and Eveline, 2011).

### Participants

Teachers were selected from three faculties with the permission of deans and on a voluntary basis. Aims, objectives, and context of the research were provided to participants, and the researchers ensured that participants are diverse through purposive sampling (i.e., gender, experience, nationality, and age). A total of 16 teachers volunteered for the interviews. Age range of participants was between 32 and 58 with 8 female teachers and 8 male. Teachers were from business, marketing, and tourism faculties, and majority (12) are married with 6 teachers having children, and had online classes with over 150 students. Furthermore, these university teachers carried out personal studies (research), lectured various classes during the week, had administrative role for handling university’s online platform for their classes, grading and exam-related tasks (e.g., preparation and management), and had advisory roles to help students (e.g., taking courses, registrations, etc.). These combined with their tutoring from home were the characteristics of sample selected for this study. These criteria were used to ensure that the participants fall within the category of university teachers, who have had their work-life balance severely impacted during COVID-19 pandemic.

### Data collection and ethics

Semi-structured interviews were held by the supervisor and first researcher during November and December 2021, in which emotions and experiences of teachers were addressed regarding wellbeing, performance, autonomy, and both personal and professional aspects. The questions of interviews were derived from existing scales as mentioned earlier. Upon conducting a pilot interview stage with two teachers from a different university, the interview setting and questions were established. Each interview was held in a place of participants’ selection (online or in-person) with an approximate of 45 min for each session. Anonymity was strictly followed when transcribing the recorded interviews, and all initial recordings were deleted after transcription and computerization of the data in MAXQDA software. Participants were also encouraged to provide any feedback or to share any ideas or thoughts that they might have regarding the topic at hand. Sharing personal information was completely arbitrary and permissions from faculty deans and university authorities were granted. Following recent and relevant works of scholars, aims and objectives of the research, theoretical framework, specification of the sample, dialog quality, and strategy for analytical techniques were taken into consideration to ensure the strength of the gathered data (Malterud et al., 2016; Varpio et al., 2017; Lases et al., 2018). The supervisor surveilled and monitored each interview to ensure that the researcher is establishing a satisfactory level of dialog quality while the first researcher conducted them. After transcription, each interviewee was asked to check the data for its correctness, to add any further comments, and to confirm whether interpretations of terms are adequate (e.g., “work on my own schedule” was coded as job autonomy).

### Reflexivity

Due to the fact that the second researcher is a teacher, it is established that the influences of researchers on the study and its conduct are restrained (Finlay, 2002; Lases et al., 2018). In this respect, a deeper sense of understanding was in place, which enabled the participants to further provide more detailed information with openness due to having an “insider” conducting the research and interviews (Asselin, 2003). However, to avoid any presumptions and or biases, the first researcher conducted the interviews so that personal presumptions are not mixed with participants’ information, and therefore, controlling the impact of personal views on findings (Fischer, 2009). Hence, while the second researcher established the notion of research with participants to provide comfort, the first researcher conducted the interviews. The researchers carefully went through each transcription and discussed the views and whether interviewers had an influence on responses or interpretations.

### Analysis

Upon completion of interviews and transcriptions, researchers reviewed the interviews several times to ensure adequate comprehension. Second author was responsible for coding the data using MAXQDA version 11 that enables researchers to analyze qualitative data. Transcriptions were coded based on their meaning, synonymy, themes, and tags. Upon assigning codes to each paragraph (concept-driven) of transcribed data and deriving themes, the researchers discussed various aspects of data and reached a unified conclusion regarding coding and interpretation of the data (e.g., wellbeing). Reliability was confirmed by “intercoder agreement” with a coefficient of 0.96. Thematic network analysis was used, in which experiences of individuals in various levels are facilitated and sorted in a manner that provides sufficient data descriptions (Lases et al., 2018). Researchers continuously discussed every aspect throughout the process of this research to ensure that the results are properly reported to reflect a better understanding of teachers’ wellbeing during COVID-19 pandemic.

## Findings

Table 1 presents the key aspects that have been extracted from the interviewees regarding their overall wellbeing. The items that are presented in Table 1 are extracted from the analysis conducted on the gathered qualitative data. In this respect, themes (left column) and specific keywords (right column) are highlighted based on the responses of interviewees as well as any synonym or terminology that was used, reflecting these items. As noted earlier, interviewees have confirmed the accuracy of terms in regard to their responses.

**Table 1:** Content analysis report.

Professional aspects	
Motivation	Knowledge deliverance, competence, job satisfaction
Engagement	Active contact with students and/or colleagues
Organizational support	Administration, information, flexibility and job autonomy, training, and HR practices
Supervisor support	Professional and personal support, job autonomy, work-family balance, and personalized care
Job autonomy	Flexible timing, customized schedules, deliverance, and reforming course structure during the semester (e.g., assignments/exams)
**Personal aspects**	
Personal characteristics	Personality, coping mechanisms, familiarity with online platforms, perceived performance
Work-life balance	Family members and friends, time management, and activities beyond work (e.g., sports),
Work–family conflict	Work-related stress and anxiety, task overload (i.e., exam period), timing and scheduling, and resource consumption (i.e., energy and time)

For instance, regarding professional aspects, respondents have provided data such as “I do not feel I have the necessary abilities to handle this state of work;” “not seeing my colleagues nor my students is taking a toll on me and how I manage my courses;” “I can work much better on my own terms and schedule;” “it helps a lot when managers are supportive and helpful. It makes a big difference when I know my managers will help me if I have any problems at home or work;” and “being in control of my courses is very helpful when I work from home because I can change or adjust things when necessary.”

Moreover, samples of what respondents provided regarding their personal aspects are, “it was relatively difficult for me to adjust to the new online system of the university;” “I did not have proper time or energy for myself to separate work and life and to take care of myself both mentally and physically;” “entertainment and leisure was not possible and I did not find other ways to make a stable routine for myself;” “working from home disrupted my normal life as it was difficult to separate different activities in the time that was needed;” “I often had to work late in the evening or night because of other responsibilities I have at home and for my family;” “I think my overall performance is much less when I work from home which makes me doubt my capabilities and I feel frustrated;” “too much pressure was on us teachers in the first periods of online teaching as we did not have proper information and some had internet issues which was very stressful;” “I was not sure how I will handle my classes from home because of my children and family. I had to make sure everything is well in home while maintaining my courses and students which put me in a constantly anxious state;” “often I felt that I have to sacrifice some responsibilities to manage other ones because of fatigue or simply lack of time;” “students were anxious and had many questions and concerns. This made me put extra hours to make sure everything is under control;” “during exam and registration period I had to work much more which was chaotic as my family duties and my life itself were diminished;” and “Not being able to see my family or friends during the lockdown was incredibly painful and at times I was so stressed that my life may lose its balance because of my work.”

## Discussion

Teachers had to spend more time (especially in the first semester that the pandemic started) on learning how to work with the newly established online systems of the university. While modules and platforms such as Pearson and Google have been used by a handful of teachers, others needed a significant amount of time to adjust to implemented systems. Notably, various participants emphasized the issues that platforms had during their early stages. Not only disconnection and lag would hinder the productivity and focus of teacher and students, such errors were reported as stressors for teachers during remote work throughout the pandemic. This was linked to other stressors such as feedback and complaints from students, losing the progress of course content, and inability to return to the session as an admin after being disconnected. Consumption of time and causing stress are key factors in this respect that are in line with described theories of this research (JD-R model, stress theories, and wellbeing; Adisa et al., 2021; Collie, 2021; Sarwar et al., 2021; Rasool et al., 2022; Syrek et al., 2022).

Feelings such as stress, anxiety, and exhaustion have led to the extraction of themes linked to burnout, sense of isolation, lack of motivation and/or engagement, and depletion of energy for other daily tasks (unrelated to work). These are key findings of the current research that while showing consensus with prior studies (e.g., Neneh, 2017; Darouei and Pluut, 2021; Nemteanu et al., 2021; Sarwar et al., 2021; Rasool et al., 2022), provide a deeper understanding on how the abrupt shift in education sector to online classes have had a psychological impact on teachers. Job autonomy and organizational support (especially supervisor support) have been repeatedly noted by participants which further supports current arguments. In this respect, young teachers have mentioned taking initiatives regarding conducting and managing online classes that significantly varied from one another. For instance, two teachers reported using online assistance through making a small group of students taking the role of mediator between teachers and the rest of the class. As a link, these groups enabled teachers to provide necessary information to students regarding important matters despite lack of adequate communication. Other academicians have used social media groups with invite link for members with a permanent link on school platforms. Furthermore, during the registration period and exam weeks, teachers have reported increased working hours which yields in more conflicts between work and life or family. While married teachers reported severity regarding the issue of time during peak periods, single participants have reported the same issues from other aspects such as not being able to spend time on their personal matters. Hence, we conclude that both life and family domains have faced conflicts during the COVID-19 pandemic for teachers. This further emphasizes the importance of job autonomy as it provides flexibility for teachers while carrying out their tasks.

Teachers have also mentioned the importance of proper supervisory actions (support and appreciation), opportunity to learn (training), and team support (colleagues) repeatedly. In this sense, various themes and keywords that are highlighted in Table 1 have been derived as vital concepts for wellbeing of teachers during the pandemic, which is linked to the extant literature (Rasool et al., 2019, 2022; Novitasari et al., 2020; Vaziri et al., 2020; Andrade and Petiz Lousã, 2021; Ghislieri et al., 2021). Notably, lack of information, functionality of online platforms for learning, and having to deal with family-related matters during work hours have been among the factors that not only caused stress and/or conflict, but further had a vivid impact on their motivation, engagement, and overall performance. This was combined with receiving complaints, and other student-related issues regarding the course and its details, which further yielded in anxiety and lowered self-esteem. In turn, these effects had a significant influence on wellbeing of teachers during this period. With other daily activities (e.g., leisure, and sports) being restricted during the pandemic, it was noted that coping strategies of teachers were diminished, and, therefore, overall wellbeing was affected in a negative manner. This was parallel with work-life balance issues such as inability to separate time and energy required for handling each domain. Teachers repeatedly mentioned the higher severity of these challenges during the first semester of the pandemic and the following summer.

While married teachers noted the conflicts of their work with their spouses and/or children, single teachers reported similar issues as they could not manage their daily activities, when forced to work from home and overcome the issues of lockdown. In this respect, stress, lack of social interaction, concerns regarding the health of self and relatives, and inadequate organizational and supervisor support were highlighted by teachers as explicit effects on their performance and wellbeing. Job autonomy can be a highly influential element in this regard. Similarly, participants revealed that their job satisfaction was reduced to a level where intentions of turnover was felt. Moreover, due to reduced engagement from students with the classes, teachers found themselves being less motivated to conduct their classes, and did not engage with material or students as they have before. As a result, motivation, job satisfaction, and perceived performance were demolished. Supervisors’ role in the provision of personalized care, information, clear tasks, flexibility and autonomy, and focus on work-life balance was noted as a significant factor. Importantly, these findings are in consensus with the extant literature (e.g., Hong et al., 2021; Sarwar et al., 2021; Oubibi et al., 2022; Syrek et al., 2022). However, a number of teachers reported poorly about their supervisors (deans) in this regard, which was a determinant during task overloads, uncertainty, and imbalance. Those who had a higher level of interactions with their peers and supervisors during this period, and were given autonomy regarding their conduct, reported a better sense of control over their work-life balance, leading to a sense of support and care for their wellbeing.

## Theoretical implications

From a theoretical view, this study was conducted within the premises of various theories that are used to imply the importance of job autonomy, support, wellbeing, and other noted elements. Within the premises of self-determination theory, job autonomy provides more motivation and personality for individuals, which current research emphasizes on as it can positively influence wellbeing of university teachers (Deci et al., 2017). Moreover, performance of individuals can greatly reduce in their roles as adequate focus and strength does not match the requirements of different roles, and inter-role conflict rises that is linked to role theory (Adisa et al., 2021). Furthermore, aforementioned notion is also linked to JD-R model as the demands of education sector are high, and work-life balance and wellbeing of university teachers are jeopardized (Sarwar et al., 2021). Due to the high demands of university, teachers’ job autonomy becomes essential as it provides them with the flexibility needed to conduct their tasks and reduce incompatibility between work and life roles. Both compensation and conflict theories have been supported in terms of their implications in the current context. Accordingly, conflicts and differences between domains of work and life are essential for performance, satisfaction, motivation, engagement, and establishing balance for university teachers (Armstrong et al., 2015; Schubert et al., 2021). Similarly, action-regulation theory is implied in this context as in the intellectual and meta-cognitive levels, individuals regulate their actions and behaviors based on their mental achievements and can enhance performance (Zacher and Frese, 2018). However, this requires an environment, where university teachers have enough autonomy, support, and are equipped with necessary tools to be able to achieve their goals. This also requires a healthy level of balance between work and life (Schubert et al., 2021). In relation with 3C theory of teacher stress, while coping strategies (e.g., sports, music, meditation, and spirituality) can vary based on individuals’ preferences, competence can be jeopardized when work and life are off balance. This, in turn, hinders performance, reduces motivation, and can have dire effects on psychological wellbeing of university teachers, leading to depression, intentions of turnover, and burnout (Herman et al., 2020; Telyani et al., 2021). However, context as the third “C”’ in this theory falls within managerial implications noted below.

## Managerial implications

In accord with current findings, we suggest that university authorities establish and implement systems of HR that provide sufficient and adequate training to teachers while taking their work-life balance into consideration. Similarly, deans as supervisors should deploy leadership styles that address wellbeing, work-life balance, and care for their followers (e.g., servant or transformational leadership; Rasool et al., 2021). These actions can improve performance, satisfaction, motivation, and aid the teachers in handling their work and life/family domains in a more profound manner. It is also important that investments are made in the IT section to enhance the online systems in terms of performance (UX and UI) to ensure that staff can easily use these tools. We emphasize the effect of job autonomy for teachers, especially during times of crisis, as it can give a leeway for teachers to schedule their classes and other tasks based on their preferred approaches. This falls within the premises of 3c theory of teacher stress in the form of context, which implies the importance of policies, methods, practices, and administrative/supervisor support.

## Limitations and recommendations

This study was limited by a number of factors, which, in turn, opened pathways for future studies. The relationship/interrelationships among the factors were not addressed in this research, which can be analyzed through PLS-SEM by future studies. In this respect, complex models can also use longitudinal data to take the variations that occur with time into account. In addition, future researchers can conduct similar studies in different sectors, which have been affected by the pandemic in a similar manner to provide comparative analysis, as this research merely focuses on teachers. Furthermore, administrative or managerial levels in education sector can be studied to better understand the apparent and latent effects. The role of HRM department was not emphasized in this study, which can be addressed by scholars conducting research on this context. Furthermore, coding and transcribing data were done by one researcher, and interviews were developed by using a number of measures from the extant literature. Scholars interested in this subject can further delegate these tasks to different researchers to decrease error term in the procedure.

## Ethics statement

Ethical review and approval were not required for the study on human participants in accordance with the local legislation and institutional requirements. Written informed consent for participation was not required for this study in accordance with the national legislation and the institutional requirements.

## Author contributions

SK: interviews and initial writing. PZ: analysis and final writing. All authors contributed to the article and approved the submitted version.

## Conflict of interest

The authors declare that the research was conducted in the absence of any commercial or financial relationships that could be construed as a potential conflict of interest.

## Publisher’s note

All claims expressed in this article are solely those of the authors and do not necessarily represent those of their affiliated organizations, or those of the publisher, the editors and the reviewers. Any product that may be evaluated in this article, or claim that may be made by its manufacturer, is not guaranteed or endorsed by the publisher.
